# Impact of pulmonary vein variant anatomy and cross-sectional orifice area on freedom from atrial fibrillation recurrence after cryothermal single-shot guided pulmonary vein isolation

**DOI:** 10.1007/s10840-022-01279-w

**Published:** 2022-06-28

**Authors:** Denise Guckel, Philipp Lucas, Khuraman Isgandarova, Mustapha El Hamriti, Leonard Bergau, Thomas Fink, Vanessa Sciacca, Martin Braun, Moneeb Khalaph, Guram Imnadze, Georg Nölker, Philipp Sommer, Christian Sohns

**Affiliations:** 1 Clinic for Electrophysiology, Herz- und Diabeteszentrum NRW, Ruhr-Universität Bochum, Bad Oeynhausen, Germany; 2 Clinic for Internal Medicine II/Cardiology, Christliches Klinikum Unna Mitte, Unna, Germany

**Keywords:** Atrial fibrillation, Catheter ablation, Cryoballoon, Single-shot ablation devices, Magnetic resonance imaging, AF recurrence

## Abstract

**Background:**

Cryoballoon (CB)-guided pulmonary vein isolation (PVI) is an established treatment for atrial fibrillation (AF). This study aimed to evaluate ablation efficacy and freedom from arrhythmia recurrence using the novel POLARx compared to the Arctic Front Advance Pro (AFA) CB system including the analysis of individual PV characteristics.

**Methods:**

A total of 687 patients underwent CB-guided ablation for AF. Arrhythmia recurrence was defined as an ECG documented episode of any AF/atrial tachycardia (AT) > 30 s. Anatomical characteristics were assessed using magnetic resonance imaging (MRI). For each PV, the cross-sectional orifice area (CSOA) was determined. Follow-up examinations were scheduled after 3, 6, and 12 months.

**Results:**

Acute PVI was achieved in all patients. Twelve-month AF-free survival was similar between the groups (POLARx 43/86 (50%) vs. AFA 318/601 (53%), Log-rank (LR) *p* = 0.346). MRI found a comparable percentage of patients with normal PV anatomy (POLARx 71/86 (83%) vs. AFA 530/601 (85%), *p* = 0.162). Patients with variant PV characteristics presented with a significantly impaired 12-month AF-free survival (normal PVs 326/585 (56%) vs. variant PVs 27/102 (27%), LR *p* < 0.001) independent of the applied CB ablation system. PAF patients with AF recurrence presented with significantly larger CSOA of the left-sided PVs and the right superior PVs (LSPV: *p* < 0.001; LIPV: *p* < 0.001; RSPV: *p* < 0.001). In PERS AF, no association between CSOA and ablation outcome was observed. Multivariate analyses identified PERS AF (hazard ratio (HR) 2.504, confidence interval (CI), 1.900–3.299, *p* < 0.001) and variant PV anatomy (HR 2.124, CI 1.608–2.805, *p* < 0.001) as independent predictors for AF recurrence.

**Conclusions:**

Both CB ablation systems are associated with comparable 12-month AF-free survival rates. Variant PV anatomy seems to be predictive for AF recurrence. An association between CSOA and the outcome after CB-guided PVI was demonstrated for PAF.

**Supplementary information:**

The online version contains supplementary material available at 10.1007/s10840-022-01279-w.

## Introduction

Cryoballoon (CB)-guided pulmonary vein isolation (PVI) is an effective treatment for atrial fibrillation (AF) [1–5]. In addition, it is an alternative to radiofrequency (RF)-guided catheter ablation, due to shorter procedure times, a steep learning curve, and a high degree of lesion reproducibility [1, 6–8]. Despite advances in catheter-based technology and operator experience, PV reconnection still remains an issue [9]. Variant PV anatomy has been discussed as a potential limitation of single-shot device-guided PVI [10, 11], but data on its impact on AF recurrence is scarce [12–17]. Recently, a second CB system (POLARx, Boston Scientific) became available. First clinical experiences with this novel system reported comparable efficacy and safety, but differences in terms of biophysiological parameters to the established system (Arctic Front Advance Pro, AFA, Medtronic) [18–21]. Data on patient outcome and the impact of individual PV characteristics on AF recurrence is lacking.

This study aimed to evaluate ablation efficacy and outcome using the novel POLARx CB compared to the established AFA system in consideration of individual anatomical characteristics and the underlying AF pattern.

## Methods

This observational study included 687 consecutive patients undergoing index CB-guided PVI for symptomatic and drug refractory paroxysmal (PAF) and persistent AF (PERS AF) between January 2013 and August 2021. No additional radiofrequency energy was applied. Data were analyzed retrospectively. Institutional standards as well as operator experience were comparable. We compared clinical characteristics and procedural outcomes of 86 patients undergoing single-shot device-guided PVI utilizing the 28-mm POLARx versus another cohort of 601 patients treated with the second-generation 28-mm AFA catheter (POLARx vs. AFA). Beyond that, we analyzed patients with a normal PV anatomy (two left- and two right-sided PVs) to patients with a variant PV anatomy (normal vs. variant PV anatomy). The diagnosis of PAF vs. PERS AF was made according to current guidelines [1]. Arrhythmia recurrence was defined as an ECG documented episode of any AF/atrial tachycardia (AT) > 30 s.

### Procedural management

Left atrium (LA)/left atrial appendage (LAA) thrombus formation was ruled out in all patients prior to ablation. Cardiac magnetic resonance imaging (MRI) was performed in all patients for procedural planning and to evaluate the individual anatomy of the LA and PVs. For each PV, the cross-sectional orifice area (CSOA) was calculated from the transversal (D1) and coronal (D2) diameters of the PVs using the equation *π* × (1/2 × D1 × D2) allowing for an approximate determination of the PV ostium dimension. PV angiography was used to check for complete occlusion of the PVs before freezing.

Antiarrhythmic drugs (AADs) except for amiodarone were discontinued at least three half-lives before ablation. Anticoagulation with phenprocoumon was continued aiming for an international normalized ratio (INR) between 2.0 and 3.0. Direct oral anticoagulants (DOAC) were stopped one half-life before ablation. Pericardial effusion (PE) was ruled out immediately after ablation and the next day. Anticoagulation was continued within 4 h after the procedure with phenprocoumon or DOAC when there was no evidence for PE. AADs were prescribed at the operators’ discretion for a period of 3 months following ablation.

### Ablation procedure

All procedures were performed under conscious sedation using propofol and analgesia with fentanyl as required. Intravenous heparin was administered to maintain an activated clotting time (ACT) of 300 s throughout the procedure.

The 28-mm AFA cryoballoon (Arctic Front Advance Pro, 8 mm tip, Medtronic) and the POLARx catheter (POLARx 5 mm or 12 mm tip, Boston Scientific) were applied. Following transseptal puncture, the balloon device was advanced into the LA via a steerable sheath (15-F FlexCath advance Medtronic or 15.9-F POLARSTEATH, Boston Scientific). A multipolar mapping catheter (Achieve Advance Mapping Catheter, Medtronic, or POLARMAP, Boston Scientific) was used for PV mapping.

Diaphragmatic excursion during ablation of the right-sided veins was confirmed via continuous stimulation of the phrenic nerve and compound motor action potential (CMAP) visualization in patients with AFA or by applying the novel diaphragm movement sensor (DMS) in patients receiving CB-guided PVI with the POALRx-system. The DMS is based on an accelerometer technology and placed on an electrode below the right-sided costal cartilage. Complete PV occlusion prior to each freezing cycle was confirmed by PV angiography with the balloon inflated and placed in the PV ostia. In all patients, ablation was performed adherent to a 2*180 s freeze per vein protocol. Persistent PVI (entrance and exit block) was confirmed after a waiting period of 20 min.

### Follow-up

After discharge, follow-up visits were scheduled 3, 6, and 12 months after the index procedure including routine 7-day Holter ECGs and interviews. Unscheduled visits were conducted if required.

### Endpoint

We aimed to evaluate ablation efficacy and outcome using the novel POLARx CB system compared to the established AFA system, to assess the impact of individual PV anatomy on freedom from arrhythmia recurrence in PAF compared to PERS AF and to compare the two ablation systems concerning individual anatomical considerations. Furthermore, we intended to ascertain independent predictors (IPs) for AF/AT recurrence in this patient cohort allowing for conclusions in terms of personalized paths in AF management. AF/AT recurrence was judged on ECG documentation and symptoms suggestive of arrhythmia recurrence.

### Data collection

Data on patients’ characteristics, medication, symptoms, and complications were compiled from patients’ records and discharge letters. Procedural parameters and clinical aspects concerning CB ablation were taken from ablation protocols and procedure-related documents.

### Statistical analysis

All statistical analyses were performed with SPSS, version 24 (SPSS, Inc., Chicago, IL, USA). All variables were tested for normal distribution. Continuous variables between the groups were compared by employing an unpaired two-sided Student’s *t*-test or Mann–Whitney test. Differences in continuous parameters between baseline and follow-up were analyzed by paired Student’s *t*-test or Wilcoxon signed-rank test. Categorical and ordinal data were examined by chi-square, Mann–Whitney tests, or Fisher’s exact tests, respectively. Event-free survival was calculated by Kaplan–Meier analysis as time from initial PVI to first documented AF episode > 30 s at the 3- and 6-month follow-ups. The log-rank test was used to assess differences in event-free survival time between groups. A Cox proportional hazard regression model was applied to identify IPs of arrhythmia recurrence. Demographic and clinical data from baseline analyses were included in univariate Cox proportional hazard regression models for the primary endpoint. Variables with an unadjusted association with AF recurrence (*p* < 0.1) were analyzed by multivariate Cox regression analysis. Data are presented as mean ± SD or percentage value unless stated otherwise. A *p*-value ≤ 0.05 was considered statistically significant.

## Results

### Patients’ characteristics

The study population consisted of 687 consecutive patients (59.5 ± 14.9 years old, 30% female) undergoing CB-guided PVI for AF. A total of 401 patients (58%) suffered from PAF (54.3 ± 15.7 years old, 29% female) and 286 patients (42%) from PERS AF (66.7 ± 10.1 years old, 30% female). Eighty-six patients (10%) were treated with the POLARx system (61.3 ± 11.1 years old, 31% female). A total of 601 patients underwent CB-guided PVI with the AFA system (59.2 ± 20.8 years old, 28% female). Depending on the applied single-shot ablation device, patients were further divided into POLARx patients with PAF (50 patients, 58%) and PERS AF (36 patients, 42%) and AFA patients with PAF (351 patients, 58%) and PERS AF (250 patients, 42%).

### Baseline characteristics (PAF versus PERS AF)

Patients with PERS AF were significantly older and more likely to have diabetes, hypertension, structural cardiomyopathy, and enlarged left atrial volume indices (LAVI) (Supplementary Table 1).

### Baseline characteristics (POLARx versus AFA)

Baseline variables were similar between the groups. Characteristics are summarized in Table 1.

**Table 1:** Baseline characteristics (POLARx versus AFA)

Characteristics	POLARx (*n* = 86)	AFA (*n* = 601)	*p*-value
Age (years)	61.3 ± 11.1	59.2 ± 20.8	0.138
Gender, female	27 (31%)	171 (28%)	0.611
BMI (kg/m^2^)	29.6 ± 8.0	27.9 ± 4.6	0.075
LVEF (%)	52.9 ± 3.7	53.5 ± 4.8	0.116
Cardiomyopathy	11 (13%)	70 (12%)	0.722
Hypertension	49 (57%)	332 (55%)	0.817
Diabetes mellitus I/II	9 (11%)	83 (14%)	0.499
Beta blocker	71 (83%)	480 (80%)	0.665
AADs	7 (8%)	76 (13%)	0.289
PAF	50 (58%)	351 (58%)	0.575
LAVI (ml/m^2^)	39.1 ± 6.7	39.2 ± 7.3	0.882

Continuous variables are shown as the mean ± SD and categorical variables as the number (%). A *p*-value ≤ 0.05 indicates statistical significance. *BMI*, body mass index; *LVEF*; left ventricular ejection fraction; *LA*, left atrium; *AADs*, antiarrhythmic agents; *PAF*, paroxysmal arterial fibrillation; *LAVI*, left atrial volume index

### Characteristics (normal versus variant PV anatomy)

Preprocedural MRI found normal LA and PV anatomy (2 left- and two right-sided PVs) in the majority of patients (*n* = 585, 85%). Variant PV anatomy was identified in 86 patients treated with AFA (14%) and in 16 patients (19%) from the POLARx cohort (left common ostium: POLARx, *n* = 8, 9%; AFA, *n* = 62, 10%; right-sided accessory vein: POLARx, *n* = 8, 9%; AFA, *n* = 14, 2%; right common ostium: AFA, *n* = 13, 2%). Between POLARx and AFA, no group-specific differences were observed (*p* = 0.162). Variant PV anatomy was equally distributed among PERS AF (*n* = 40, 16%) and PAF (*n* = 62, 15%) (*p* = 0.912).

### Procedural data (POLARx versus AFA)

POLARx group patients presented with a significantly longer procedure duration (POLARx: 113.9 ± 23.4 min vs. AFA: 100.7 ± 32.5 min, *p* < 0.001) and fluoroscopy time (POLARx: 10.9 ± 7.1 min vs. AFA: 8.4 ± 7.5 min, *p* = 0.010). The POLARx balloon achieved significantly lower nadir temperatures during the freeze application in all PVs. Details are summarized in Table 2 and Supplementary Table 2.

**Table 2:** Procedural data

Characteristics	POLARx (*n* = 86)	AFA (*n* = 601)	*p*-value
Total procedure time (min)	113.9 ± 23.4 [95.0, 130.0]	100.7 ± 32.5 [85.0, 113.5]	< 0.001
Total fluoroscopy time (min)	10.9 ± 7.1 [3.4, 12.5]	8.4 ± 7.5 [2.5, 13.0]	0.010
Contrast agent (ml)	39.4 ± 12.4 [30.0, 46.5]	40.5 ± 17.1 [20.0, 55.0]	0.410
Cumulative radiation dose (cGycm^2^)	426.5 ± 630.2 [117.0, 442.7]	543.2 ± 372.7 [241.0, 703.0]	0.316
LSPV Nadir temperature (°C)	− 58.1 ± 5.2 [− 61.0, − 55.0]	− 45.9 ± 5.8 [− 49.0, − 43.0]	< 0.001
LIPV Nadir temperature (°C)	− 57.0 ± 5.4 [− 60.0, − 53.0]	− 42.1 ± 4.3 [− 44.8, − 39.0]	< 0.001
LCV Nadir temperature (°C)	N/A	− 41.0 ± 9.6 [− 39.5, − 45.8]	
RSPV Nadir temperature (°C)	− 58.4 ± 6.4 [− 62.0, − 53.0]	− 47.4 ± 6.7 [− 44.07, − 42.3]	< 0.001
RIPV Nadir temperature (°C)	− 55.3 ± 7.8 [− 63.0, − 54.8]	− 45.8 ± 7.2 [− 51.0, − 41.0]	< 0.001

Continuous variables are shown as the mean ± SD and as median [25^th^ and 75.^th^ percentiles]. A *p*-value ≤ 0.05 indicates statistical significance. *LSPV*, left superior pulmonary vein; *LIPV*, left inferior pulmonary vein; *LCV*, left common pulmonary vein; *RIPV*, right inferior pulmonary vein; *RSPV*, right superior pulmonary vein

### Clinical outcome

Acute PVI was achieved in all patients (*n* = 687, 100%). AF recurrence was detected in 326 patients (47%) within the follow-up period. A total of 7% (*n* = 48) was lost to follow-up. Baseline and procedural data of those patients with partially lacking data were compared to those with complete data sets and no significant differences were found.

#### POLARx versus AFA

**Fig. 1:**
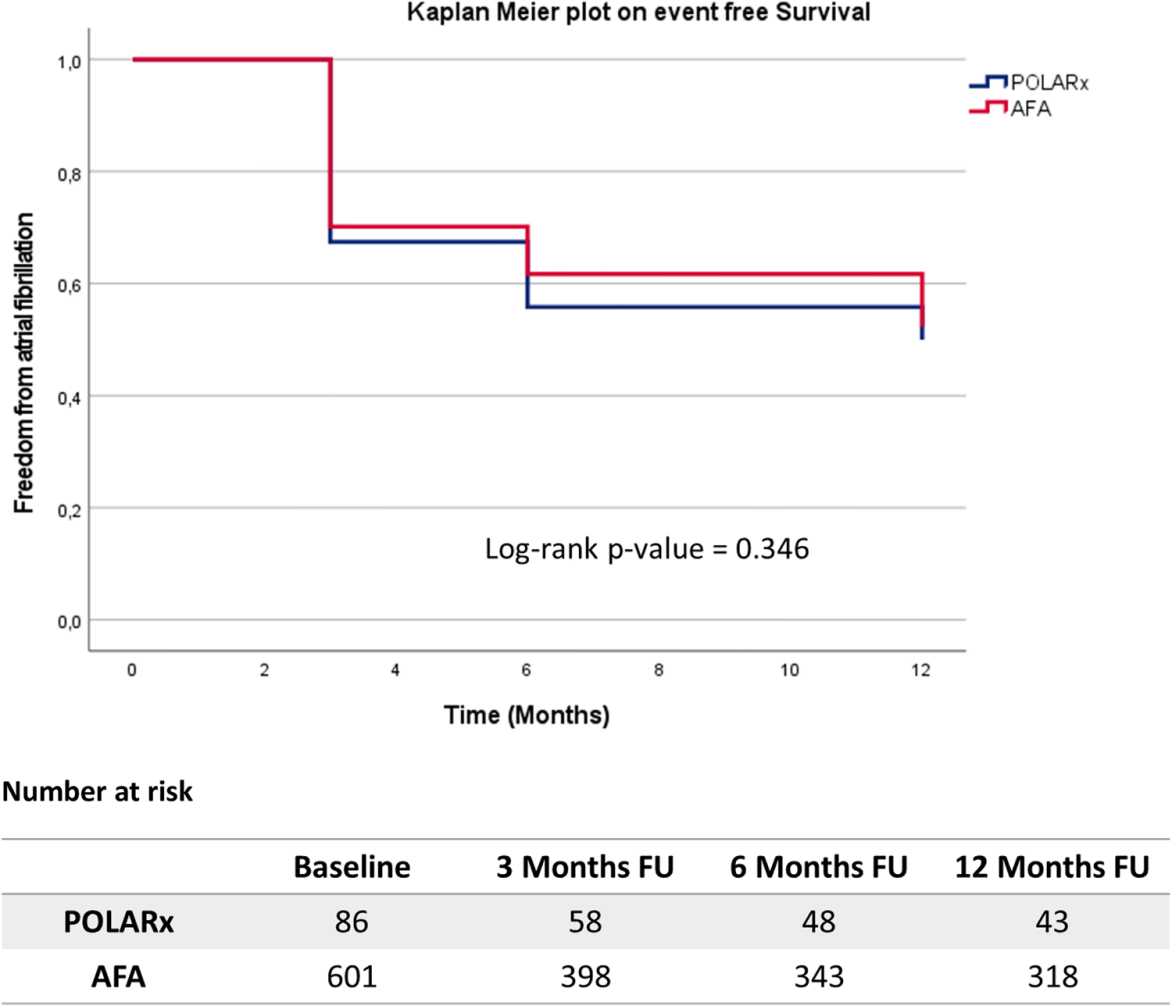
Kaplan–Meier analysis of freedom from AF recurrence in patients undergoing CB-guided PVI (POLARx vs. AFA). A *p*-value ≤ 0.05 indicates statistical significance. AF, atrial fibrillation; CB, cryoballoon; PVI, pulmonary vein isolation; PV, pulmonary vein; FU, follow-up

The estimated 12-month AF-free survival was comparable between both groups (POLARx: *n* = 43, 50%; AFA: *n* = 318, 53%; Log-rank *p* = 0.346; Fig. 1.

#### PAF versus PERS AF

Patients with PERS AF presented with significantly higher recurrence rates compared to patients diagnosed with PAF (PERS AF: *n* = 204, 71% vs. PAF: *n* = 122, 30%, *p* < 0.001). Kaplan–Meier analyses revealed a comparable estimated AF-free survival between POLARx- and AFA-treated patients including PAF and PERS AF. Details are presented in Supplementary Fig. 1.

### Clinical outcome depending on PV anatomy

**Fig. 2:**
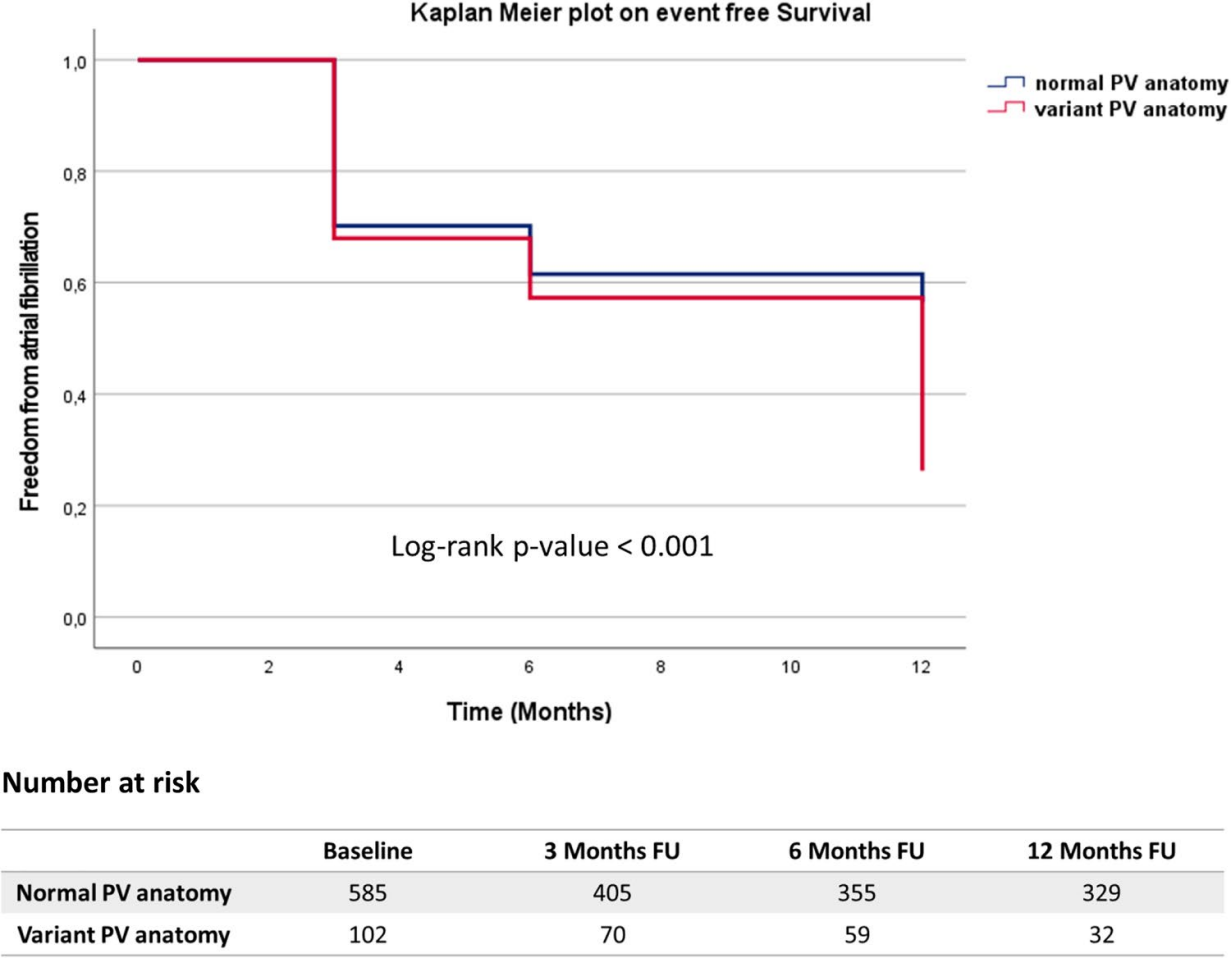
Kaplan–Meier analysis of freedom from AF recurrence in patients undergoing CB-guided PVI (normal PV vs. variant PV). A *p*-value ≤ 0.05 indicates statistical significance. AF, atrial fibrillation; CB, cryoballoon; PVI, pulmonary vein isolation; PV, pulmonary vein; FU, follow-up

Patients with normal PV anatomy presented with significantly lower AF recurrence rates compared to patients with variant PV characteristics (normal PV: *n* = 256, 44% vs. variant PV: *n* = 70, 69%, *p* < 0.001); Fig. 2. The AF recurrence rate in patients with a left common pulmonary vein (LCV) amounted to 36% (*n* = 25).

#### POLARx versus AFA

In both groups, MRI revealed a comparable percentage of patients with normal PV anatomy (POLARx: *n* = 70, 81% vs. AFA, *n* = 515, 86%, *p* = 0.329). Focusing on AF-free survival, patients with normal PV characteristics presented with a significantly improved outcome in the POLARx as well as in the AFA cohort.

#### PAF versus PERS AF

Focusing on AF-free survival, significant differences were observed in PAF patients depending on individual PV characteristics (normal PV: 245, 82% vs. variant PV: 17, 33%, log-rank *p* < 0.001), whereas no significant differences were observed in PERS AF (normal PV: 93, 43% vs. variant PV: 6, 18%, Log-rank *p* = 0.134).

### Impact of cross-sectional orifice area (CSOA) on AF recurrence

#### POLARx versus AFA

In terms of PV occlusion, no significant CSOA-related differences were observed in patients with AF recurrence and those without. Detailed information is shown in Table 3.

**Table 3:** Impact of CSOA on AF recurrence (POLARx versus AFA)

POLARx (*n* = 86)	AF recurrence (*n* = 43)	No AF recurrence (*n* = 43)	*p*-value
LSPV	190.4 ± 45.1 [153.2, 228.7]	189.0 ± 66.9 [137.2, 224.9]	0.929
LIPV	181.9 ± 50.6 [141.5, 225.2]	165.2 ± 43.4 [131.9, 202.1]	0.163
RSPV	235.0 ± 78.5 [174.6, 285.5]	237.4 ± 90.0 [163.8, 276.4]	0.908
RIPV	257.1 ± 132.9 [178.2, 294.5]	230.5 ± 106.8 [158.7, 292.6]	0.355
AFA (*n* = 601)	AF recurrence (*n* = 283)	No AF recurrence (*n* = 318)	*p*-value
LSPV	225.4 ± 74.56 [175.9, 268.6]	222.8 ± 61.2 [176.7, 254.5]	0.739
LIPV	152.4 ± 68.7 [109.9, 188.5]	146.5 ± 57.7 [110.0, 176.7]	0.401
RSPV	262.9 ± 80.9 [208.9, 313.4]	268.5 ± 77.1 [213.2, 325.5]	0.499
RIPV	239.7 ± 86.6 [183.8, 282.7]	239.4 ± 68.9 [197.9, 268.6]	0.966

Continuous variables are shown as the mean ± SD and as median [25^th^ and 75.^th^ percentiles]. A *p*-value ≤ 0.05 indicates statistical significance. *CSOA*, cross-sectional orifice area; *AF*, atrial fibrillation; *LSPV*, left superior pulmonary vein; *LIPV*, left inferior pulmonary vein; *RIPV*, right inferior pulmonary vein; *RSPV*, right superior pulmonary vein

#### PAF versus PERS AF

No differences in terms of PV CSOA were revealed between patients with PERS AF and PAF, but CSOA was a predictor for AF recurrence in patients with PAF. Patients with PAF and AF recurrence have had significantly larger CSOA of the left-sided and the right superior PVs compared to patients without arrhythmia recurrence. In PERS AF, no relationship between CSOA and freedom from AF recurrence was observed. Details are summarized in Table 4.

**Table 4:** Impact of CSOA on AF recurrence (PAF versus PERS AF)

PAF (*n* = 401)	AF recurrence (*n* = 126)	No AF recurrence (*n* = 275)	*p*-value
LSPV	242.8 ± 88.1 [162.0, 273.1]	221.5 ± 63.5 [176.4, 254.3]	< 0.001
LIPV	154.7 ± 61.2 [91.7, 184.3]	141.2 ± 53.9 [107.2, 170.3]	< 0.001
RSPV	269.2 ± 79.3 [222.4, 302.4]	258.2 ± 73.4 [202.5, 302.7]	< 0.001
RIPV	253.7 ± 103.5 [192.3, 267.1]	232.1 ± 70.2 [187.2, 283.4]	0.220
PERS AF (*n* = 286)	AF recurrence (*n* = 208)	No AF recurrence (***n* = 78)**	*p*-value
LSPV	231.2 ± 65.2 [187.4, 263.2]	226.7 ± 59.1 [174.5, 267.5]	0.218
LIPV	155.9 ± 69.1 [116.7, 196.7]	156.2 ± 65.1 [116.7, 199.3]	0.587
RSPV	262.3 ± 86.2 [209.7, 313.3]	277.2 ± 74.2 [217.9, 330.2]	0.523
RIPV	233.6 ± 81.5 [182.7, 267.2]	251.2 ± 76.2 [202.3, 284.2]	0.544

Continuous variables are shown as the mean ± SD and as median [25^th^ and 75.^th^ percentiles]. A *p*-value ≤ 0.05 indicates statistical significance. *PAF*, paroxysmal atrial fibrillation; *PERS AF*, persistent atrial fibrillation; *LSPV*, left superior pulmonary vein; *LIPV*, left inferior pulmonary vein; *RIPV*, right inferior pulmonary vein; *RSPV*, right superior pulmonary vein

From left atrial volume index (LAVI), no relevant differences were found between patients with normal PV dimensions and those with enlarged CSOAs (normal PV dimensions 38.076 ± 6.136 versus enlarged CSOAs 38.555 ± 7.420 ml/m^2^, *p* = 0.360).

### Predictors for AF recurrence

Multivariate Cox regression analysis identified variant PV anatomy (hazard ratio (HR) 2.124, confidence interval (CI) 1.608–2.805, *p* < 0.001) and PERS AF (HR 2.504, CI 1.900–3.299, *p* < 0.001) as IP for AF recurrence. Details are presented in Supplementary Table 3.

### Complications

In the AFA group, a periprocedural thromboembolic event was observed in one patient (< 1%), and two patients suffered from PE requiring pericardiocentesis (< 1%), persistent phrenic nerve palsy (< 1%), and vascular groin complications (< 1%). In the POLARx group, PE requiring treatment occurred in one patient (2%). In both groups, no esophageal perforation/fistula or death occurred.

## Discussion

### Main findings

This study has three major findings: First, the POLARx and the AFA systems are associated with comparable 12-month AF-free survival rates. Second, variant PV anatomy seems to be predictive for AF recurrence. Third, in PAF, a correlation between AF recurrence and an enlarged CSOA was observed for the left-sided and the right superior PV.

### Impact of the applied ablation system on AF-free survival

In contrast to the AFA system, the POLARx offers a stable size and equal balloon pressure during the inflation and ablation period. This might help to prevent slight shifts of the balloon during the freezing cycle to achieve an adequate balloon-to-tissue contact [19]. Irrespective of these technical innovations, PV occlusion seems to be comparably effective for both systems resulting in almost equal AF-free survival rates (Fig. 1).

### Impact of PV characteristics on freedom from AF recurrence

#### CSOA

Two smaller studies reported that there might be a relevant impact of the CSOA on freedom from AF recurrence following ablation [10, 13]. This acts in concert with our observation that patients with PAF and an enlarged CSOA of the left-sided and/or right superior PV have had a higher rate of AF recurrence (Table 4). Enlarged PVs and the related CSOA might impede complete electrical isolation of the PVs and inadequate isolation or lesion formation increases the risk for PV reconnection. In a prior study, we documented that the presence of an enlarged PV (LCV) is associated with more freeze cycles for complete PVI [22]. Larger PVs seem to require more freeze applications for complete PVI. Beyond that, enlarged PVs may contribute to an individual arrhythmia substrate that promotes AF recurrence [8, 13]. Apart from enlarged PV dimensions, some studies identified the superior PVs as the most common site of origin for ectopic beats potentially initiating AF [14]. In PERS AF, PV characteristics seem to play a minor role most likely due to advanced stages of atrial fibrosis and scar formation (Table 4).

A prior study suggested a critical relationship between structural alterations of LA tissue and AF initiation addressing that PV dimensions seem to have a greater impact on AF recurrence than the pure size of the LA [23] and these findings are in line with our observations. Thus, preprocedural imaging and the estimation of the CSOA may help to identify patients who benefit most from single-shot device-guided PVI.

#### Variant PV anatomy

Variant PV anatomy has been reported to influence AF recurrence after RF-guided PVI [16]. This observation might be explained due to limited catheter-to-tissue contact force and difficulties in obtaining durable transmural lesions [16]. Kubala et al. reported on 118 patients scheduled for CB-guided PVI where normal PV anatomy was associated with an improved outcome, particularly in PAF [8]. In our cohort, variant PV anatomy was present in 15% of patients and thus comparable to previous studies [12]. In addition, variant PV anatomy seemed to be predictive for AF recurrence independent of the applied ablation system with more pronounced effects in PAF. In contrast, the recent STOP-AF trial (*n* = 163 patients) [17] and Wei et al. (*n* = 424 patients) [12] identified variant PV anatomy as irrelevant for freedom from AF recurrence following CB-guided PVI and Bose et al. as well as Heeger et al. reported that a LCV was not predictive for AF recurrence, too [24, 25].

### Procedural data

We suppose a learning curve effect as a reason for longer procedure and fluoroscopy times (Table 2 and Supplementary Table 2) which has also been reported and discussed previously [20]. In addition, our data is in line with previous reports indicating that PVI using the POLARx catheter achieves significantly lower nadir temperatures [18–20]. The optimal minimum temperature for the POLARx system seems to be about − 5 to − 10 °C lower compared to the established system [18–20]. This could be explained by differences in material properties, expansion pressure, or a slightly different position of the temperature probe within the novel POLARx system. As the POLARx catheter offers a stable size and equal balloon pressure during the inflation and ablation period, it might help to prevent from any kind of pop-out phenomenon as well as slight shifts of the balloon during the freezing cycle. Thus, an exact coaxial alignment and only minimal push are required to achieve an adequate balloon-to-tissue contact. Beyond that, the handling of the POLARx system is comparably smart and straightforward due to improved material properties. The POLARSTEATH appears to be softer and more flexible [22].

Irrespective of these differences in nadir temperature, quality of PV occlusion and procedural success were comparable between the two ablation devices. At the same time, complication rates were comparably low, which is in line with previous data [18–20].

The YETI registry found an incidence of 4.2% for phrenic nerve injury [26]. In our study, phrenic nerve injury was very rare (< 1%), and consequently, no specificities in terms of variable PV anatomy could be identified.

### Limitations

The present study is of observational design and therefore has certain limitations. The POLARx cohort is relatively small. Nevertheless, our POLARx group is one of the largest cohorts of patients who underwent PVI utilizing the novel cryothermal balloon device so far. A potential but improbable bias by missing follow-up data in the AFA cohort of patients (7%, *n* = 48) cannot be excluded. For each PV, the CSOA of the PVs was calculated using the equation *π* × (1/2 × D1 × D2) allowing for an approximate, not an exact, determination of the PV ostium dimension. The lack of the exact PV area as an indicator to differentiate the CBs may be relevant, and perhaps, it is not the PV area that characterizes differences between the CBs. Therefore, and as both CBs are able to compensate to a certain degree for individual anatomical deviations, the approximate determination of the PV ostium dimensions appears to be adequate and sufficient to address the objectives of this manuscript.

## Conclusion

CB-guided ablation with both single-shot systems is associated with comparable 12-month AF-free survival rates. Variant PV anatomy seems to be relevant for AF recurrence. An association between CSOA and the outcome after CB-guided PVI is documented for PAF.

## Supplementary Information

Below is the link to the electronic supplementary material.

Supplementary file1 (PDF 417 KB)

## References

[1] Hindricks G, Potpara T, Dagres N, Arbelo E, Bax JJ, Blomström-Lundqvist C, Boriani G, Castella M, Dan GA, Dilaveris PE, Fauchier L, Filippatos G, Kalman JM, La Meir M, Lane DA, Lebeau JP, Lettino M, Lip GYH, Pinto FJ, Thomas GN, Valgimigli M, Van Gelder IC, Van Putte BP, Watkins CL; ESC Scientific Document Group.Hindricks G, et al. 2020 ESC Guidelines for the diagnosis and management of atrial fibrillation developed in collaboration with the European Association for Cardio-Thoracic Surgery (EACTS): The Task Force for the diagnosis and management of atrial fibrillation of the European Society of Cardiology (ESC) Developed with the special contribution of the European Heart Rhythm Association (EHRA) of the ESC. Eur Heart J. 2021 ;42:373–498. 10.1093/eurheartj/ehaa612 32860505

[2] Calkins H, Hindricks G, Cappato R, Kim YH, Saad EB, Aguinaga L, Akar JG, Badhwar V, Brugada J, Camm J, Chen PS, Chen SA, Chung MK, Cosedis Nielsen J, Curtis AB, Davies DW, Day JD, d'Avila A, Natasja de Groot NMS, Di Biase L, Duytschaever M, Edgerton JR, Ellenbogen KA, Ellinor PT, Ernst S, Fenelon G, Gerstenfeld EP, Haines DE, Haissaguerre M, Helm RH, Hylek E, Jackman WM, Jalife J, Kalman JM, Kautzner J, Kottkamp H, Kuck KH, Kumagai K, Lee R, Lewalter T, Lindsay BD, Macle L, Mansour M, Marchlinski FE, Michaud GF, Nakagawa H, Natale A, Nattel S, Okumura K, Packer D, Pokushalov E, Reynolds MR, Sanders P, Scanavacca M, Schilling R, Tondo C, Tsao HM, Verma A, Wilber DJ, Yamane T, Document Reviewers: Calkins H,  (2017). HRS/EHRA/ECAS/APHRS/SOLAECE expert consensus statement on catheter and surgical ablation of atrial fibrillation. Europace.

[3] Guckel D, Schmidt A, Gutleben KJ, Körber B, Fischbach T, Horstkotte D, Sommer P, Nölker G.Guckel D, et al. Pulmonary vein isolation and beyond: Predictive value of vagal reactions in second-generation cryoballoon ablation for the outcome of persistent atrial fibrillation. Heart Rhythm. 2020;17:600–606. 10.1016/j.hrthm.2019.12.006 31841715

[4] Bergau L, El Hamriti M, Rubarth K, Dagher L, Molatta S, Braun M, Khalaph M, Imnadze G, Nölker G, Nowak CP, Fox H, Sommer P, Sohns C.Bergau L, et al. Cool enough? Lessons learned from cryoballoon-guided catheter ablation for atrial fibrillation in young adults. J Cardiovasc Electrophysiol. 2020;31:2857–2864. 10.1111/jce.14717 33345455

[5] Sohns C, Marrouche NF, Costard-Jäckle A, Sossalla S, Bergau L, Schramm R, Fuchs U, Omran H, Rubarth K, Dumitrescu D, Konietschke F, Rudolph V, Gummert J, Sommer P, Fox H.Sohns C, et al. Catheter ablation for atrial fibrillation in patients with end-stage heart failure and eligibility for heart transplantation. ESC Heart Fail. 2021;8:1666–1674. 10.1002/ehf2.13150 PMC8006697 33314690

[6] Kuck KH, Brugada J, Furnkranz A, Metzner A, Ouyang F, Chun KR, Elvan A, Arentz T, Bestehorn K, Pocock SJ, Albenque JP, Tondo C; FIRE AND ICE Investigators. Cryoballoon or radiofrequency ablation for paroxysmal atrial fibrillation. N Engl J Med 2016; 374:2235_2245. 10.1056/NEJMoa1602014 27042964

[7] Luik A, Radzewitz A, Kieser M, Walter M, Bramlage P, Hormann P, Schmidt K, Horn N, Brinkmeier-Theofanopoulou M, Kunzmann K, Riexinger T, Schymik G, Merkel M, Schmitt C (2015). Cryoballoon versus open irrigated radiofrequency ablation in patients with paroxysmal atrial fibrillation: the prospective, randomized, controlled, noninferiority FreezeAF study. Circulation.

[8] Kubala M, Hermida JS, Nadji G, Quenum S, Traulle S, Jarry G (2011). Normal pulmonary veins anatomy is associated with better AF-free survival after cryoablation as compared to atypical anatomy with common left pulmonary vein. Pacing Clin Electrophysiol.

[9] McLellan AJ, Ling LH, Ruggiero D, Wong MC, Walters TE, Nisbet A, Shetty AK, Azzopardi S, Taylor AJ, Morton JB, Kalman JM, Kistler PM (2014). Pulmonary vein isolation: the impact of pulmonary venous anatomy on long-term outcome of catheter ablation for paroxysmal atrial fibrillation. Heart Rhythm.

[10] Neuzil P, Reddy VY, Kautzner J, Petru J, Wichterle D, Shah D, Lambert H, Yulzari A, Wissner E, Kuck KH (2013). Electrical reconnection after pulmonary vein isolation is contingent on contact force during initial treatment: results from the EFFICAS I study. Circ Arrhythm Electrophysiol.

[11] Kumar S, Morton JB, Lee J, Halloran K, Spence SJ, Gorelik A, Hepworth G, Kistler PM, Kalman JM (2012). Prospective characterization of catheter-tissue contact force at different anatomic sites during antral pulmonary vein isolation. Circ Arrhythm Electrophysiol.

[12] Wei HQ, Guo XG, Zhou GB, Sun Q, Yang JD, Xie HY, Zhang S, Liang JJ, Ma J.J Procedural findings and clinical outcome of second-generation cryoballoon ablation in patients with variant pulmonary vein anatomy. Cardiovasc Electrophysiol. 2019 ;30:32–38. 10.1111/jce.13768 30288848

[13] Nakashima T, Kawasaki M, Toyoshi H, Takasugi N, Kubota T, Kanamori H, Ushikoshi H, Aoyama T, Nishigaki K, Minatoguchi S (2018). Impact of the pulmonary vein orifice area assessed using intracardiac echocardiography on the outcome of radiofrequency catheter ablation for atrial fibrillation. J Interv Card Electrophysiol.

[14] Lin WS, Prakash VS, Tai CT, Hsieh MH, Tsai CF, Yu WC, Lin YK, Ding YA, Chang MS, Chen SA (2000). Pulmonary vein morphology in patients with paroxysmal atrial fibrillation initiated by ectopic beats originating from the pulmonary veins: implications for catheter ablation. Circulation.

[15] Sohns C, Sohns JM, Bergau L, Sossalla S, Vollmann D, Lüthje L, Staab W, Dorenkamp M, Harrison JL, O'Neill MD, Lotz J, Zabel M (2013). Pulmonary vein anatomy predicts freedom from atrial fibrillation using remote magnetic navigation for circumferential pulmonary vein ablation. Europace.

[16] Hunter RJ, Ginks M, Ang R, Diab I, Goromonzi FC, Page S, Baker V, Richmond L, Tayebjee M, Sporton S, Earley MJ, Schilling RJ (2010). Impact of variant pulmonary vein anatomy and image integration on long-term outcome after catheter ablation for atrial fibrillation. Europace.

[17] Andrade JG, Khairy P, Macle L, Packer DL, Lehmann JW, Holcomb RG, Ruskin JN, Dubuc M (2014). Incidence and significance of early recurrences of atrial fibrillation after cryoballoon ablation: insights from the multicenter Sustained Treatment of Paroxysmal Atrial Fibrillation (STOP AF) Trial. Circ Arrhythm Electrophysiol.

[18] Creta A, Kanthasamy V, Schilling RJ, Rosengarten J, Khan F, Honarbakhsh S, Earley MJ, Hunter RJ, Finlay M (2021). Creta A, et al First experience of POLARx versus Arctic Front Advance: An early technology comparison. J Cardiovasc Electrophysiol.

[19] Tilz RR, Meyer-Saraei R, Eitel C, Fink T, Sciacca V, Lopez LD, Kirstein B, Schlüter M, Vogler J, Kuck KH, Heeger CH.Tilz RR, et al. Novel Cryoballoon Ablation System for Single Shot Pulmonary Vein Isolation - The Prospective ICE-AGE-X Study. Circ J. 2021;85:1296–1304. 10.1253/circj.CJ-21-0094 33854004

[20] Kochi AN, Moltrasio M, Tundo F, Riva S, Ascione C, Dessanai MA, Pizzamiglio F, Vettor G, Cellucci S, Gasperetti A, Tondo C, Fassini G.Kochi AN, et al. Cryoballoon atrial fibrillation ablation: Single-center safety and efficacy data using a novel cryoballoon technology compared to a historical balloon platform. J Cardiovasc Electrophysiol. 2021;32:588–594. 10.1111/jce.14930 33537996

[21] Anic A, Lever N, Martin A, Breskovic T, Sulkin MS, Duffy E, Saliba WI, Niebauer MJ, Wazni OM, Varma N.Anic A, et al. Acute safety, efficacy, and advantages of a novel cryoballoon ablation system for pulmonary vein isolation in patients with paroxysmal atrial fibrillation: initial clinical experience. Europace. 2021;23:1237–1243. 10.1093/europace/euab018 PMC8350865 33729470

[22] Guckel D, Lucas P, Isgandarova K, El Hamriti M, Bergau L, Fink T, Sciacca V, Imnadze G, Braun M, Khalaph M, Nölker G, Sommer P, Sohns C (2022). News from the Cold Chamber: Clinical Experiences of POLARx versus Arctic Front Advance for Single-Shot Pulmonary Vein Isolation. J Cardiovasc Dev Dis.

[23] Rettmann ME, Holmes DR 3rd, Monahan KH, Breen JF, Bahnson TD, Mark DB, Poole JE, Ellis AM, Silverstein AP, Al-Khalidi HR, Lee KL, Robb RA, Packer DL; CABANA Imaging Investigators. Treatment-Related Changes in Left Atrial Structure in Atrial Fibrillation: Findings From the CABANA Imaging Substudy. Circ Arrhythm Electrophysiol. 2021;14:e008540. 10.1161/CIRCEP.120.008540 PMC8193460 33848199

[24] Bose A, Chevli PA, Berberian G, Januszkiewicz J, Ahmad G, Hashmath Z, Mishra AK, Laidlaw D (2021). Presence of a left common pulmonary vein and pulmonary vein anatomical characteristics as predictors of outcome following cryoballoon ablation for paroxysmal atrial fibrillation. J Interv Card Electrophysiol.

[25] Heeger CH, Tscholl V, Wissner E, Fink T, Rottner L, Wohlmuth P, Bellmann B, Roser M, Mathew S, Sohns C, Reißmann B, Lemeš C, Maurer T, Santoro F, Riedl J, Goldmann B, Landmesser U, Ouyang F, Kuck KH, Rillig A, Metzner A (2017). Acute efficacy, safety, and long-term clinical outcomes using the second-generation cryoballoon for pulmonary vein isolation in patients with a left common pulmonary vein: A multicenter study. Heart Rhythm.

[26] Heeger CH, Sohns C, Pott A, Metzner A, Inaba O, Straube F, Kuniss M, Aryana A, Miyazaki S, Cay S, Ehrlich JR, El-Battrawy I, Martinek M, Saguner AM, Tscholl V, Yalin K, Lyan E, Su W, Papiashvili G, Botros MSN, Gasperetti A, Proietti R, Wissner E, Scherr D, Kamioka M, Makimoto H, Urushida T, Aksu T, Chun JKR, Aytemir K, Jędrzejczyk-Patej E, Kuck KH, Dahme T, Steven D, Sommer P, Richard TR (2022). Phrenic Nerve Injury During Cryoballoon-Based Pulmonary Vein Isolation: Results of the Worldwide YETI Registry. Circ Arrhythm Electrophysiol.

