# Supplementary material for: Impact of pulmonary vein variant anatomy and cross-sectional orifice area on freedom from atrial fibrillation recurrence after cryothermal single-shot guided pulmonary vein isolation
Source: J Interv Card Electrophysiol. 2022 Jun 28;65(1):251–60. doi: 10.1007/s10840-022-01279-w (PMC9550688; doi:10.1007/s10840-022-01279-w)
Supplement: Supplementary file 1 — Supplementary file1 (PDF 417 KB) [file 10840_2022_1279_MOESM1_ESM.pdf]

**Supplementary Table 1 Baseline Characteristics (PAF versus PERS AF)**

| Characteristics | PAF (n=401) | PERS AF (n=286) | p-value |
|---|---|---|---|
| Age (years) | 54.3 ± 15.7 | 66.7 ± 10.1 | < 0.001 |
| Gender, female | 118 (29%) | 85 (30%) | 0.933 |
| BMI (kg/m$^2$) | 27.7 ± 6.0 | 29.5 ± 5.1 | < 0.001 |
| LVEF (%) | 54.0 ± 3.8 | 53.6 ± 3.7 | 0.143 |
| Cardiomyopathy | 36 (9%) | 45 (16%) | 0.008 |
| Hypertension | 181 (45%) | 197 (70%) | < 0.001 |
| Diabetes mellitus I/II | 28 (7%) | 60 (21%) | < 0.001 |
| LAVI (ml/m$^2$) | 37.8 ± 7.6 | 40.1 ± 7.1 | < 0.001 |

Continuous variables are shown as the mean±SD and categorical variables as the number (%). A p-value ≤0.05 indicates statistical significance. PAF, paroxysmal arterial fibrillation, PERS AF, persistent atrial fibrillation, BMI, body mass index, LVEF, left ventricular ejection fraction, LAVI, left atrial volume index.

**Supplementary Table 2 Procedural parameter depending on a Learning Phase**

| | POLARx (n=86) | | p-value |
|---|---|---|---|
| | learning phase (n=15) | after learning phase (n=71) | |
| Total procedure time (min) | 116.5 ± 20.4 [99.0,131.0] | 111.1 ± 23.0 [95.3,126.8] | 0.389 |
| Total fluoroscopy time (min) | 10.8 ± 4.1 [7.6, 13.0] | 8.7 ± 2.9 [6.5, 10.7] | 0.083 |
| | AFA (n=601) | | p-value* |

| | | | |
|---|---|---|---|
| Total procedure time (min) | NA | 100.7 ± 32.5 [85.0,113.5] | 0.874 |
| Total fluoroscopy time (min) | NA | 8.4 ± 7.5 [2.5, 13.0] | 0.746 |

Continuous variables are shown as the mean ± SD and as median [25th and 75th percentiles]. A p-value ≤0.05 indicates statistical significance. * p-value (POLARx after learning phase vs. AFA after learning phase)

**Supplementary Table 3 Multivariate Cox-Regression Analysis**

| Variables | Hazard Ratio | Confidence Interval | p-value |
|---|---|---|---|
| AFA | 1.018 | 0.721-1.437 | 0.921 |
| PERS AF | 2.504 | 1.900-3.299 | < 0.001 |
| BMI (kg/m$^2$) | 1.014 | 0.997-1.031 | 0.119 |
| LVEF (%) | 0.997 | 0.965-1.033 | 0.855 |
| Cardiomyopathy | 1.050 | 0.741-1.487 | 0.784 |
| Hypertension | 1.233 | 0.946-1.606 | 0.121 |
| Diabetes mellitus I/II | 0.841 | 0.599-1.182 | 0.320 |
| Gender, male | 1.013 | 0.781-1.314 | 0.922 |
| Variant PV anatomy | 2.124 | 1.608-2.805 | < 0.001 |
| Age (years) | 1.016 | 1.006-1.027 | 0.327 |
| LAVI (ml/m$^2$) | 1.008 | 0.992-1.023 | 0.335 |

A p-value ≤0.05 indicates statistical significance. PERS AF, persistent atrial fibrillation; BMI, body mass index; LVEF, left ventricular ejection fraction, PV, pulmonary vein, LAVI, left atrial volume index.

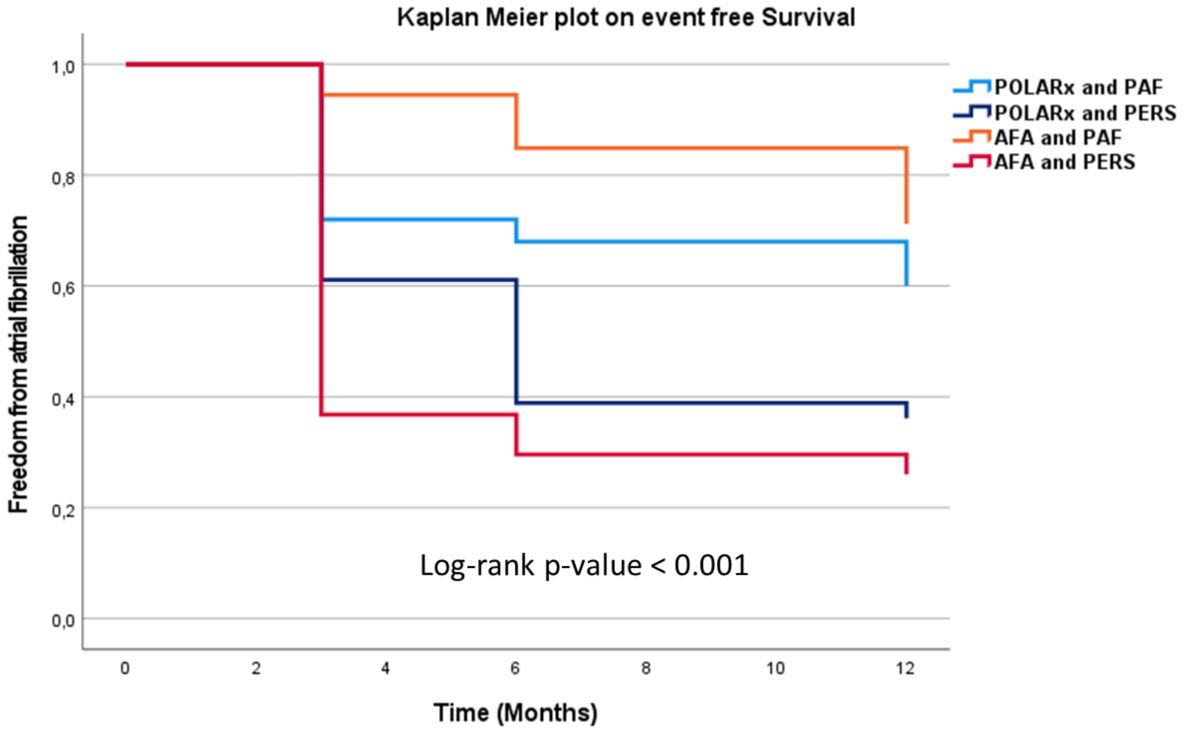


**Number at risk**

| | Baseline | 3 Months FU | 6 Months FU | 12 Months FU |
|---|---|---|---|---|
| **POLARx and PAF** | 50 | 36 | 34 | 30 |
| **POLARx and PERS** | 36 | 22 | 14 | 13 |
| **AFA and PAF** | 351 | 325 | 292 | 249 |
| **AFA and PERS** | 250 | 92 | 74 | 69 |

**Supplementary Figure 1.** Kaplan Meier plot on freedom from AF recurrence in patients undergoing CB-guided PVI due to AF (POLARx versus AFA and PAF versus PERS). A p-value ≤ 0.05 indicates statistical significance. AF, atrial fibrillation; CB, cryoballoon; PVI, pulmonary vein isolation; PAF, paroxysmal atrial fibrillation; PERS, persistent atrial fibrillation; FU, follow-up.
